# Extracellular and Intracellular Mechanisms of Mechanotransduction in Three-Dimensionally Embedded Rat Chondrocytes

**DOI:** 10.1371/journal.pone.0114327

**Published:** 2014-12-05

**Authors:** Suguru Shioji, Shinji Imai, Kosei Ando, Kousuke Kumagai, Yoshitaka Matsusue

**Affiliations:** Department of Orthopedic Surgery, Shiga University of Medical Science, Otsu, Shiga, Japan; University of Maryland School of Medicine, United States of America

## Abstract

**Purpose:**

Articular cartilage homeostasis involves modulation of chondrocyte matrix synthesis in response to mechanical stress (MS). We studied extracellular and intracellular mechanotransduction pathways mediating this response.

**Methods:**

We first confirmed rapid up-regulation of the putative chondro-protective cytokine, interleukin (IL)-4, as an immediate response to MS. We then studied the role of IL-4 by investigating responses to exogenous IL-4 or a specific IL-4 inhibitor, combined with MS. Next we investigated the intracellular second messengers. Since chondrocyte phenotype alters according to the extracellular environment, we characterized the response to mechanotransduction in 3-dimensionally embedded chondrocytes.

**Results:**

Expression of aggrecan and type II collagen was significantly up-regulated by exogenous IL-4 whereas MS-induced matrix synthesis was inhibited by an IL-4 blocker. Further, MS-induced matrix synthesis was completely blocked by a p38 MAPK inhibitor, while it was only partially blocked by inhibitors of other putative second messengers.

**Conclusion:**

IL-4 mediates an extracellular pathway of mechanotransduction, perhaps *via* an autocrine/paracrine loop, while p38 mediates an intracellular pathway prevalent only in a 3-dimensional environment.

## Introduction

Articular cartilage covers the ends of bones within joints, enabling them to move smoothly over one another. Chondrocytes maintain articular cartilage homeostasis by altering matrix synthesis in response to mechanical stress (MS). Although cell-matrix interactions are pivotal in mediating MS, the detailed mechanism regulating chondrocyte metabolism remains obscure. However, it is likely to depend on molecules such as cytokines in the immediate environment. Interleukin (IL)-1 and tumor necrosis factor-α (TNFα), both pro-inflammatory cytokines, are produced during cartilage repair and up-regulate metalloproteinase expression [1], while inflammation-induced cartilage degradation is counteracted by “cartilage-protective” cytokines [2], including IL-4, IL-10, and IL-13 [3]–[5].

It has been shown that mechanical stress (MS) on human articular chondrocytes leads to release of IL-4 [6]. Articular chondrocytes increases aggrecan synthesis in response to mechanical stimulation, which was blocked by IL-4 antibody [7]. Normal and osteoarthritic chondrocytes have been shown to express the IL-4 receptor [7], [8]. According to our review of literature, however, these studies have used monolayer-culture chondrocytes and it remains yet unclear whether IL-4 is produced by differentiated chondrocytes *in vivo* [9].

De-differentiated chondrocytes under pre-OA condition may not respond to a variety of changes in the cellular and extracellular environments such as mechanical stress. Exogenous application of cytokines and/or modulation of second messengers mediating MS-induced matrix synthesis may provide new opportunities for the therapeutic modality of osteoarthritis.

Since chondrocyte metabolic activity responds to the extracellular environment, we studied chondrocytes embedded in a three-dimensional (3D) collagen gel. First, cells were subjected to MS, and matrix synthesis was evaluated by reverse transcription (RT)-PCR for type II collagen (Col2) and aggrecan (AGC). We then investigated whether IL-4 can re-activate chondrocytes which have de-differentiated through monolayer proliferation, and found that IL-4 indeed up-regulated both Col2 and AGC expression. In turn, MS increased IL-4 expression, suggesting that IL-4 acts as a paracrine/autocrine signaling molecule during mechanotransduction.

Various pathways have been implicated in chondrocyte signaling following mechanical activation, including mitogen activated protein kinases (MAPKs), which are responsible for the conversion of many extracellular stimuli into specific cellular responses ranging from modulation of cell proliferation, differentiation, and apoptosis to regulation of inflammatory and stress responses [10]–[13]. Three major MAPK families - ERK1 and ERK2 [14]–[16], JNK [10], [17], [18], and p38 [14], [19] - have been identified in mammalian cells, but their roles in chondrocyte mechanotransduction remain controversial and may reflect different extracellular environments. We characterized both extracellular and intracellular signaling cascades by which MS regulates the metabolism of 3D-embedded chondrocytes. Putative signaling pathways were blocked using specific inhibitors.

## Materials and Methods

All of the experimental protocols conform to The Guide for the Care and Use of Laboratory Animals published by the US National Institutes of Health (NIH Publication no. 85–23, revised 1996) and were approved by the Animal Care and Use Committee of Shiga University of Medical Science (09-162).

### Isolation of chondrocytes

Chondrocytes were isolated from hip, knee, and shoulder articular cartilage of seven 5-week-old Wistar rats as previously described [20], yielding approximately 5.0×10^5^ chondrocytes per rat.

### 3D embedded chondrocytes

The suspended cells were seeded at 1.0×10^5^ cells/100 mm dish and cultured in monolayer in a humidified atmosphere of 5% CO_2_ at 37°C, with medium changes every 3 days. Cells from each animal were cultured separately (n = 7). Immediately after reaching confluence, the cells were trypsinized and combined with type I collagen gel to a final density of 1.0×10^6^ cells/mL as described previously [20], and 0.2 mL of this mixture was placed in a chamber. These embedded cells were cultured in SF medium with L-ascorbic acid (50 µg/mL) for 24 hours, then subjected to real-time RT-PCR.

### Mechanical stimulation

We previously demonstrated that 1 h of MS with 5% strain most effectively enhanced AGC and Col2 expression [20], and then, the histological analysis demonstrated that the cells mechanically-stressed for 1 h were characterized by a larger oval cell soma, suggesting the most active proteoglycan production [20]. After 24 h cultured in SF medium, 3D-embedded chondrocytes were stressed using the Cell Stretcher System NS 500 (Scholar Tech, Osaka, Japan) as previously described [20].

For RT-PCR study, 3D-embedded chondrocytes were stressed for 60 min/day whereas non-stressed (NS) cells served as controls. Mechanical loading took the form of cyclic compression, and the amplitude and frequency of compression followed previous studies, which adjusted to 5% strain [21] and 0.33 Hz [22]. Gels were loaded with a cyclic stress of 2.35 kilopascals (kPa) at peak stress (1 kPa  = 1.0×10^3^ N/m^2^). Immediately after mechanical loading, chondrocytes were analyzed as described below.

### Experimental protocol 1

Experimental groups were: IL-4 (–), MS (–); IL-4 (–), MS (+); IL-4 (+), MS (–); IL-4 (+), MS (+). MS was applied at 5% strain. IL-4 (Sigma, Saint Louis, MO) was added at 10 ng/mL (n = 7). Immediately after 60 min of MS, the gel-embedded cells were removed for total RNA extraction and real-time RT-PCR.

### Experimental protocol 2

The IL-4 soluble receptor (sIL-4R, R&D Systems) was used to assess the effect of IL-4 on 3D-embedded chondrocytes, while the second messenger pathways of ERK, JNK and p38 were investigated using the specific inhibitors UO126 [23]–[25], SP600125 [17], [18], [23], and SB203580 [26]–[28], respectively, prior to applying MS. Immediately after 60 min of mechanical loading, total RNA was extracted from the embedded cells for real-time RT-PCR.

### RNA extraction and reverse transcription

Total RNA was extracted using the NucleoSpin RNA L kit (Macherey-Nagel, Düren, Germany) according to the manufacturer's protocol, eluted in PCR-grade water, and stored at −80°C. RNA was reverse transcribed into single-stranded cDNA as previously described [20]; cDNA was eluted in PCR-grade water and stored at −30°C.

### Quantitative real-time RT-PCR

Real-time RT-PCR was performed with the LightCycler System (Roche Diagnostics) using LightCycler FastStart DNA Master Hybridization Probes (Roche Diagnostics) following the manufacturer's protocol. Each reaction was performed in a 20-µL mixture containing 5 µL of cDNA and 15 µL master mix. Specific primers and probes were used for aggrecan (AGC), type II collagen (Col2), and glyceraldehyde-3-phosphate dehydrogenase (GAPDH) (Nihon Gene Laboratories Inc., Sendai Miyagi, Japan) and for IL-4 (NGRL, Miyagi, Japan).

### Statistical analysis

Results are expressed as means with 95% confidence intervals (C.I.). Differences between groups were analyzed by one-factor analysis of variance (ANOVA) with Bonferroni/Dunn post hoc tests. Differences between means were considered statistically significant at *P*-values <0.05.

## Results

### Effects of mechanical loading on 3D-embedded chondrocytes

**Figure 1:**
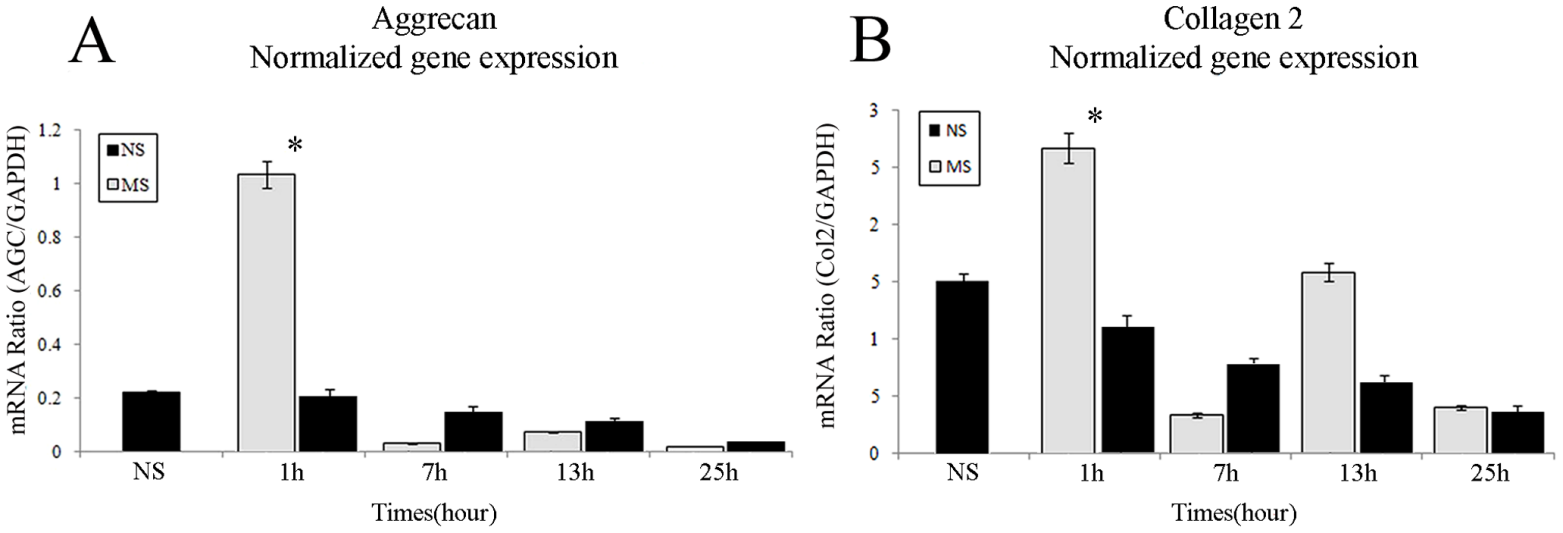
Mechanical stress-induced up-regulation of aggrecan and type II collagen. Effects of MS (60 min/day) on relative expression of (A) aggrecan (AGC), and (B) type II collagen (Col2) by chondrocytes in a 3D matrix were assessed at 1, 7, 13, and 25 h by real-time RT-PCR. Results are expressed as mean (95% C.I.), n = 7. Means were compared by one-factor ANOVA. *P<0.05 versus NS.

Expression of AGC and Col2 was measured at 1, 7, 13 and 25 h after the start of optimal MS application. Expression of AGC was significantly enhanced at 1 h (Fig. 1A), but was then rapidly down-regulated and remained low during the studied period (Fig. 1A). Thus, MS activated AGC expression in 3D-embedded chondrocytes, but this activation terminated once the stimulus was removed. In turn, Col2 expression underwent bimodal down-regulation following the initial up-regulation at 1 h (Fig. 1B). The detailed mechanistic links involved in MS-induced matrix synthesis were then studied.

### Effects of IL-4 on 3D-embedded chondrocytes

**Figure 2:**
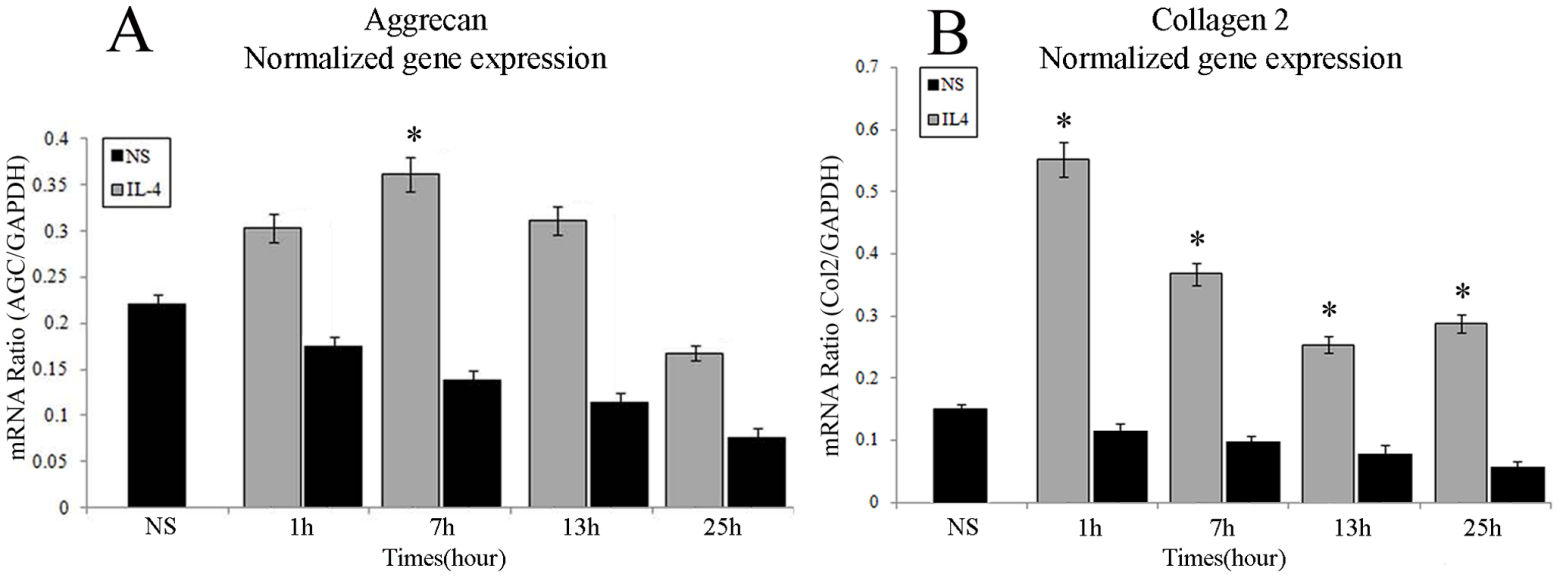
Interleukin-4-induced up-regulation of aggrecan and type II collagen. Effects of IL-4 on relative expression of (A) AGC, and (B) Col2 by chondrocytes in a 3D matrix with 10 ng/mL IL-4 were assessed at 1, 7, 13, and 25 h by real-time RT-PCR. Results are expressed as mean (95% C.I.), n = 7. Means were compared by one-factor ANOVA. *P<0.05 versus NS.

To study the role of IL-4 in regulation of the matrix synthesis, we added IL-4 to 3D-embedded chondrocytes without applying mechanical stress. AGC and Col2 expression was measured at 1, 7, 13 and 25 h after addition of IL-4 (10 ng/mL). IL-4 significantly enhanced expression of AGC, which peaked at 7 h (Fig. 2A), returning to control levels by 25 h (Fig. 2A). For Col2, expression was increased 5- to 6-folds, which peaked at 1 h (Fig. 2B), whereas AGC enhancement remained below 2-fold (Fig. 2A).

### IL-4 expression by 3D-embedded chondrocytes

**Figure 3:**
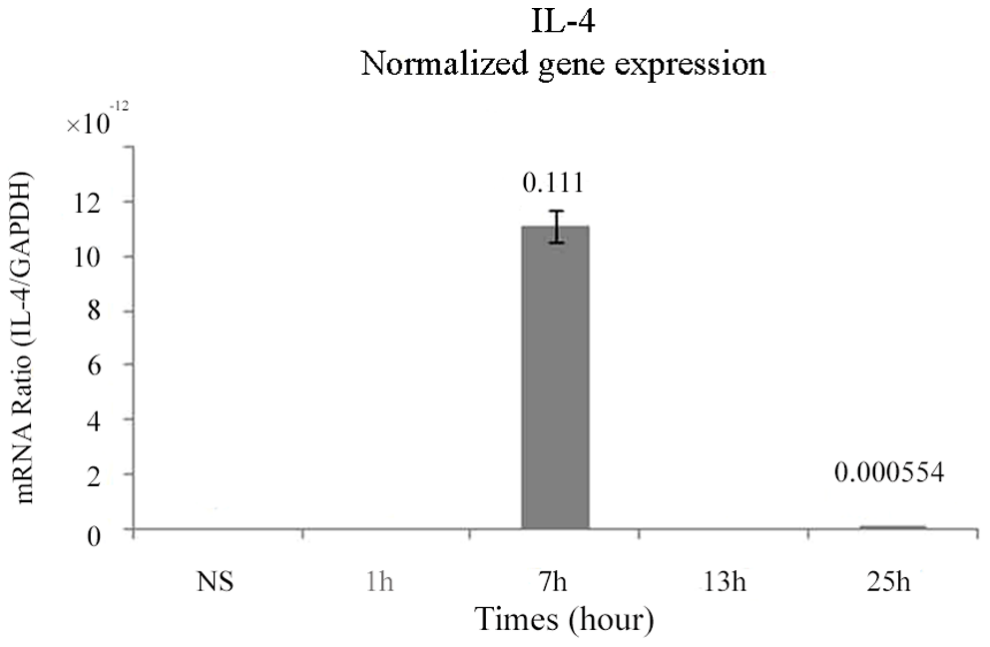
Mechanical stress-induced up-regulation of interleukin-4 gene expression. IL-4 gene expression by 3D-embedded, mechanically stressed (60 min/day), chondrocytes was analyzed by real time RT-PCR.

IL-4 has been shown to be present in monolayer chondrocytes [6], [7], [9]. On the other hand, Rai et al. revealed that utilized IL-4 in a 3D cartilage model in the previous study [29]. They transfected dog chondrocytes with IL-4 and showed that IL-4 down-regulated messenger RNA of IL-1 beta and others under simulated inflammatory condition, but they did not report endogenous expression of IL-4 by 3D-embedded chondrocytes. So, we concluded that there have been no previous studies demonstrating that MS enhances expression IL-4 using 3D-embedded chondrocytes. To test whether MS-induced matrix synthesis requires IL-4 in the 3D environment, we studied IL-4 expression in mechanically-stimulated chondrocytes using real-time RT-PCR. In agreement with our hypothesis, the 3D-embedded chondrocytes up-regulated IL-4 expression after dynamic compression. Expression was almost entirely limited to the first 7 h, being barely detectable at 13 and 25 h (Fig. 3).

### Effects of IL-4 inhibitors on 3D-embedded chondrocytes under mechanical stress

**Figure 4:**
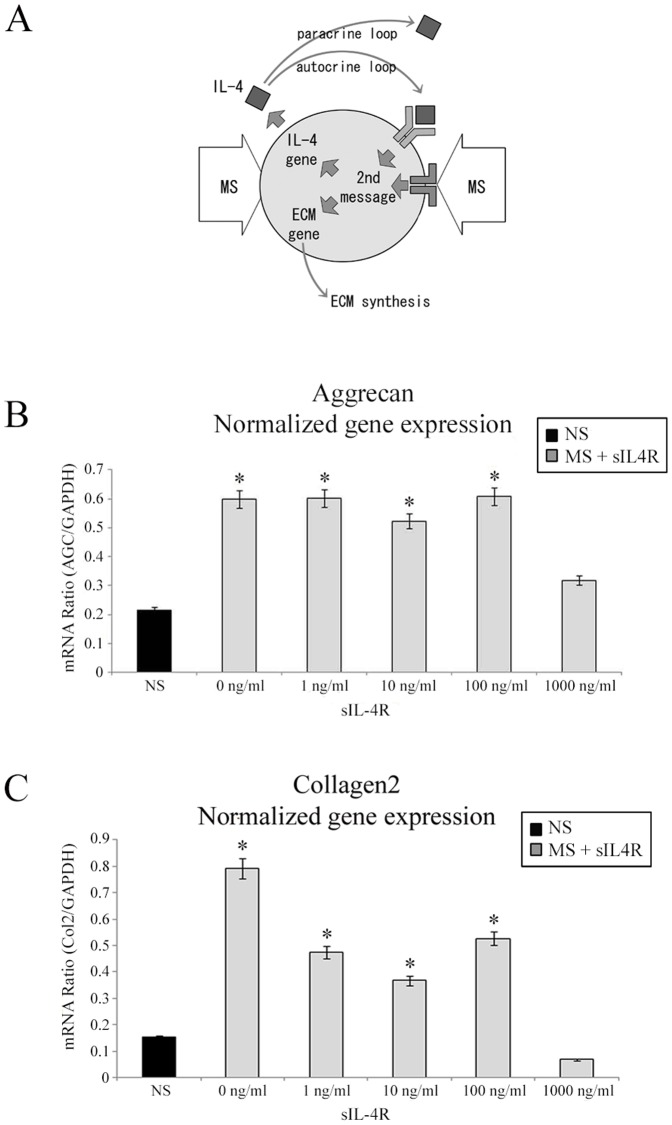
Inhibition of mechanical stress-induced up-regulation of aggrecan and type II collagen by adding soluble IL-4 receptor. (A) Schematic diagram showing putative mechanotransduction pathways in 3D-embedded chondrocytes following mechanical stimulation. (B) Effect of IL-4 inhibitors on 3D-embedded chondrocytes during mechanical loading. Dynamic compressive loading (60 min/day) was applied in combination with soluble IL-4 receptor (sIL-4R) at a range of concentrations (1, 10, 100, and 1000 ng/mL). (C) Effect of soluble IL-4 receptor (sIL-4R) on type II collagen (Col2) gene expression. All data are shown as mean (relative to GAPDH, 95% C.I.). n = 7. *P<0.05 versus NS by one-factor ANOVA.

Conditioned medium from mechanically-stimulated chondrocytes affects the membrane potential of naïve chondrocytes via IL-4 [6], while we found that mechanically stimulated 3D-embedded chondrocytes up-regulate IL-4 expression. To test whether IL-4 is involved in mechanotransduction we used the IL-4 soluble receptor (sIL-4R) to block putative effects of an IL-4 loop (Fig. 4A). One hour after applying MS, sIL-4R was added to the medium at various concentrations ranging from 1 ng/mL to 1000 ng/mL. Because enhancement of IL-4 requires 7 h (Fig. 3), AGC and Col2 mRNA were measured at 7 h after the initial application of MS.

MS-induced enhancement of AGC mRNA was not affected by sIL-4R at any concentrations but at the highest concentration of 1000 ng/mL sIL-4R (Fig. 4B). MS-induced enhancement of Col2 mRNA was similarly inhibited (Fig. 4C), supporting the existence of a paracrine/autocrine IL-4 signaling loop (Fig. 4A).

### Effects of MAPK inhibitors on MS-induced chondrocyte activation

**Figure 5:**
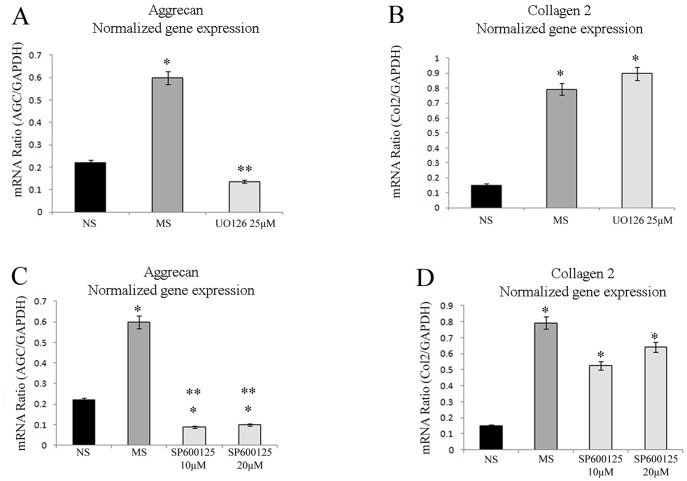
Effects of MAPK inhibitors on aggrecan and type II collagen expression during mechanical loading. (A) Effect of the ERK pathway inhibitor UO126 (25 µM) on AGC expression. (B) Effect of UO126 on Col2 expression. (C) Effect of the JNK inhibitor SP600125 on AGC expression at different concentrations (10, 20 µM). (D) Effect of SP600125 on Col2 expression. All data are shown as relative means (95% C.I.), n = 7. * *P*<0.05 versus NS, ** *P*<0.05 versus MS by one-factor ANOVA.

MS-induced activation of matrix synthesis is thought to occur *via* second messenger cascades (Fig. 4A). Various pathways including mitogen activated protein kinase (MAPK) pathways have been implicated in this signaling process [10]–[13] but it is unclear which plays the major role. To investigate this, the extracellular-regulated kinase (ERK1/2) pathway was first blocked with UO126, a specific inhibitor of ERK1/2 [23]–[25], in the 3D-embedded chondrocytes. UO126 clearly inhibited the MS-induced up-regulation of AGC expression (Fig. 5A), but it did not interfere with the MS-induced activation of Col2 (Fig. 5B), suggesting that Col2 activation is independent of ERK1/2-dependent signaling in this system.

Next, the c-Jun N-terminal kinase (JNK) pathway was blocked using the specific inhibitor SP600125 [17], [18], [23], which clearly inhibited MS-induced activation of AGC expression (Fig. 5C) but did not interfere with the MS-induced enhancement of Col2 (Fig. 5D). It suggests that Col2 activation is also independent of the JNK-dependent signaling pathway in 3D-embedded chondrocytes.

**Figure 6:**
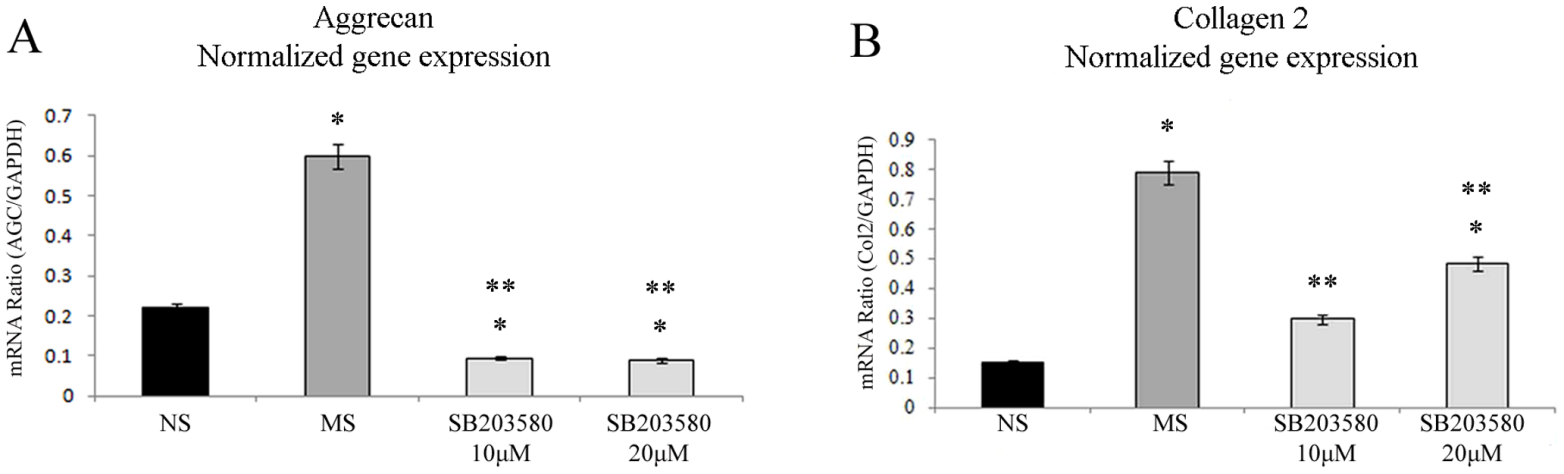
Effects of mechanical stress and the p38 inhibitor SB203580 on aggrecan and type II collagen mRNA expression during mechanical loading. (A) Effect of MS combined with the p38 inhibitor SB203580 on AGC gene expression at different concentrations (10, 20 µM). (B) Effect of the p38 inhibitor SB203580 on Col2 gene expression. All data are shown as relative means (95% C.I.), n = 7. * *P*<0.05 versus NS, ** *P*<0.05 versus MS by one-factor ANOVA.

Since inhibition of both the ERK1/2 pathway and the JNK pathway interfered with the MS-induced activation of AGC expression, both pathways were assumed to participate in the AGC response to MS, but were unlikely to mediate the Col2 response. To identify the pathway implicated in MS-induced activation of Col2, the p38 MAPK pathway was blocked by using the specific inhibitor SB203580 [26]–[28], which significantly inhibited MS-induced activation of both AGC and Col2 genes (Fig. 6A, B). All of the MAPK inhibitors were added prior to MS and total RNA was extracted immediately after MS. One should remember that up-regulation of IL-4 requires 7 h after MS (Fig. 3). Therefore, these findings suggest that the p38 MAPK signaling pathway mediates the MS-induced activation of ECM synthesis, but not the downstream IL-4 effects.

## Discussion

Chondrocytes alter their metabolic activities according to their mechanical environment. Cellular responses to MS *in vitro* in monolayer cultures have been studied using methods including compressive strain, tensile strain and hydrostatic pressure. However, this cellular environment differs from that *in vivo*, and it has been classically known that monolayer-cultured chondrocytes de-differentiate and decrease matrix synthesis [30]–[32].

In contrast, primary chondrocyte culture embedded in collagen gel could proliferate, synthesize the ECM molecules and maintain the chondrocyte phenotype for up to 4 weeks [33]. Some used different scaffolds such as alginate [34] or agarose [35] and demonstrated various effects on phenotype-preservation of chondrocytes. Others used type I collagen as scaffold for 3D culture of chondrocytes and had demonstrated its phenotype-preserving property [36]–[37]. Atelocollagen (Koken, Tokyo, Japan) is a type I collagen construct used in these previous studies [36]–[37] as well as in the present and our previous study [20].

In our previous study [20], we applied a cyclic compression with its amplitude and frequency adjusted to 5% strain [21] and 0.33 Hz [22] to result in most effective enhancement of AGC and Col2 expression [20]. Because elasticity of different 3D construct may result in altered strain of cells, the present study used Atelocollagen and followed the compression protocol adjusted in the previous study [20].

We analyzed matrix synthesis in chondrocytes 3D-embedded in a collagen matrix by evaluating expression of the chondrocyte-specific markers Col2 and AGC. As expected, the 3D-embedded chondrocytes increased matrix synthesis in response to MS.

For the first time, we demonstrate that 3D-embedded chondrocytes up-regulate IL-4 expression under MS (Fig. 3). Moreover, in accordance with our hypothesis, synthesis of the matrix components Col2 and AGC by 3D-embedded chondrocytes was significantly enhanced by IL-4 (Fig. 2A, B). These observations further suggest that chondrocytes may share information on their mechanical environment *via* an autocrine/paracrine loop involving IL-4 (Fig. 4A). Application of IL-4 to unstressed chondrocytes might therefore exert “cartilage-protective” effects by replicating the response to MS.

It has been shown that STAT signaling is implicated with IL-4 activation [9]. However, mechanical stress results in AGC and Col2 up-regulation in 1 h, while IL-4 up-regulation requires 7 h. We assume that MS does not act only through IL-4, but it does lead to paracrine communication among chondrocytes by IL-4 in parallel (Fig. 4A). Thus, we further studied second messengers related chondrogenesis and matrix synthesis. Previous studies have suggested important roles for the ERK, JNK and p38 MAP kinases in chondrogenesis in response to MS. However, the effects of MS on activation of ERK, one of the second messengers of the MAPK pathways, have been reported in several types of cells [11], [38] but both positive and negative roles have been reported in chondrocytes [24], [25]. Likewise, controversial reports have been published on the JNK-dependent increase in proteoglycan synthesis in response to cyclical mechanical strain [18], [39]. Many of the previous studies were conducted using chondrocytes cultured in monolayer. It is well-known that chondrocytes de-differentiate in the monolayer environment [31]–[33]. The controversial results concerning second messengers pertinent to mechanotransduction could therefore be attributed to the various degrees of chondrocyte de-differentiation in monolayer culture.

In the present report, we demonstrate that application of a p38 inhibitor to 3D-embedded chondrocytes significantly inhibits MS-induced activation of both AGC and Col2 genes, suggesting that the p38 MAPK signaling pathway plays an important role in MS-induced activation of 3D-embedded chondrocytes.

It has been shown that de-differentiation of chondrocytes due to a pre-OA condition may result in an inability to respond to changes in the cellular environment, including MS. Future studies on normalization or enhancement of cellular responses, e.g., gene transduction of IL-4, may provide new opportunities for the therapeutic modality of osteoarthritis.
